# Sirtuins in the Cardiovascular System: Potential Targets in Pediatric Cardiology

**DOI:** 10.1007/s00246-018-1848-1

**Published:** 2018-03-02

**Authors:** Alessandro Ianni, Xuejun Yuan, Eva Bober, Thomas Braun

**Affiliations:** Department of Cardiac Development and Remodeling, Max-Planck-Institute for Heart and Lung Research, Ludwig Strasse 43, 61231 Bad Nauheim, Germany

**Keywords:** Sirtuins, Heart, Pediatric cardiology, CHD

## Abstract

Cardiovascular diseases represent a major cause of death and morbidity. Cardiac and vascular pathologies develop predominantly in the aged population in part due to lifelong exposure to numerous risk factors but are also found in children and during adolescence. In comparison to adults, much has to be learned about the molecular pathways driving cardiovascular diseases in the pediatric population. Sirtuins are highly conserved enzymes that play pivotal roles in ensuring cardiac homeostasis under physiological and stress conditions. In this review, we discuss novel findings about the biological functions of these molecules in the cardiovascular system and their possible involvement in pediatric cardiovascular diseases.

## Sirtuins: Biological Functions

**Fig. 1:**
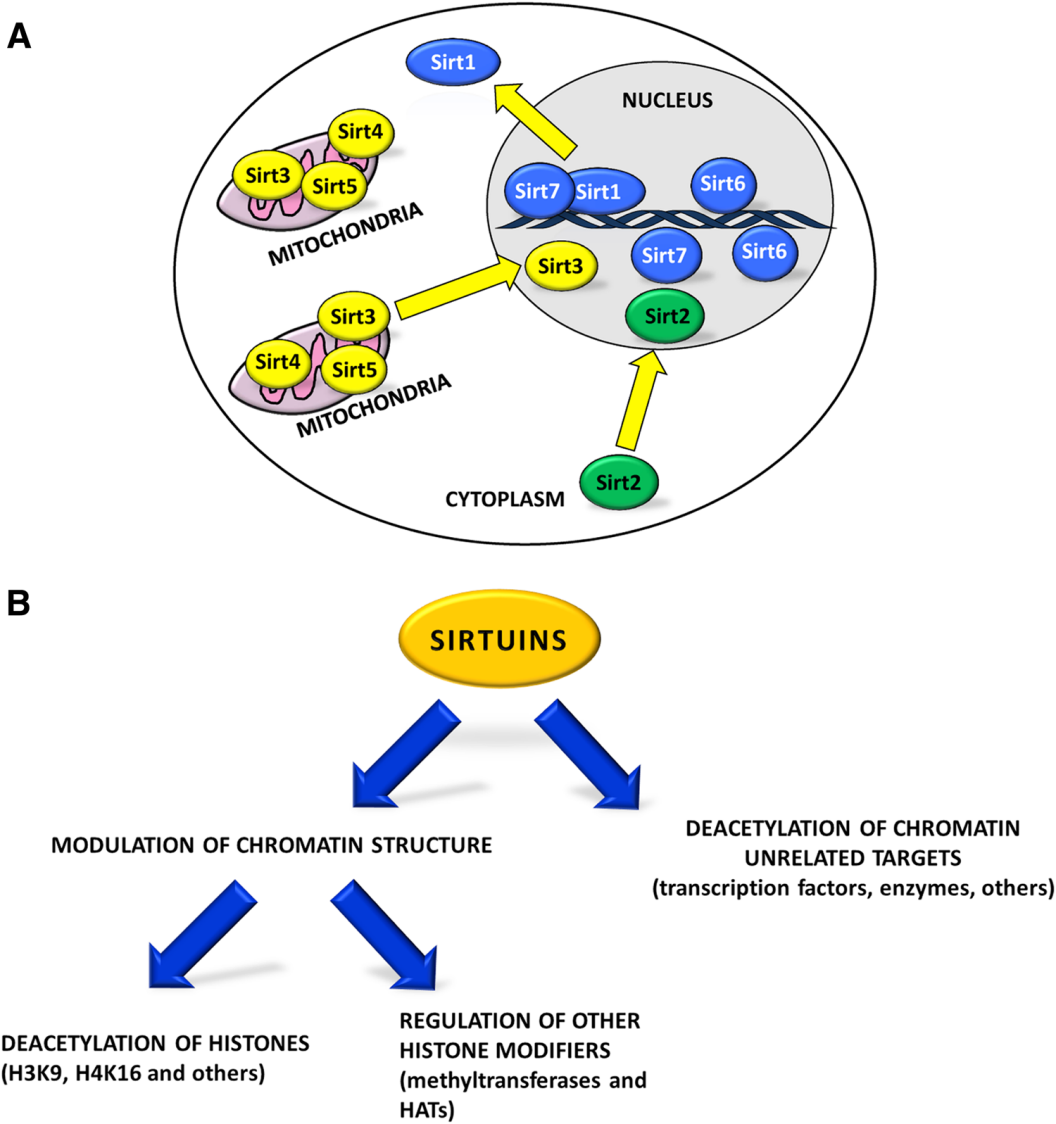
Subcellular localization and function of sirtuins. **a** Subcellular localization of the seven mammalian sirtuins. Sirt1, Sirt6, and Sirt7 are mainly localized in the nucleus. Sirt2 is a cytoplasmic enzyme while Sirt3, Sirt4, and Sirt5 are enriched in mitochondria [7]. In response to internal and external stimuli, sirtuins can translocate into other cellular compartments (yellow arrows) regulating a vast number of biological functions [8–11]. **b** Sirtuins control chromatin dynamics either by directly deacetylating histones such as histone 3 at lysine 9 (H3K9) and lysine 16 of histone 4 (H4K16) or through regulation of other histone modifiers such as methyltransferases and histone acetyltransferases (HATs). In addition, sirtuins regulate, mainly through direct deacetylation, several other targets such as transcription factors and enzymes [18]

Sirtuins constitute a class of highly conserved enzymes, which act preferentially as NAD^+^-dependent deacetylases and/or mono-ADP-ribosyl transferases. In addition, some sirtuins exhibit less characterized enzymatic activities such as desuccinylation, glycohydrolase, and demalonylation [1–5]. In mammals, sirtuins comprise a family of seven members (Sirt1-Sirt7; Fig. 1a). Mammalian sirtuins share a conserved catalytic domain but possess different N-terminal and C-terminal sequences, which are unique for each member of the family and are important for their subcellular localization and isoform-specific functions [1, 6]. Sirt1, Sirt6, and Sirt7 are mainly present in the nucleus; Sirt2 primarily resides in the cytoplasm while Sirt3, Sirt4, and Sirt5 are mitochondrial enzymes [7]. Despite their preferential localization, sirtuins can shuttle between different cellular compartments in response to internal or external cues [8–11]. Mammalian sirtuins are implicated in numerous biological processes such as cellular differentiation, metabolism, cancer progression, apoptosis, maintenance of genomic stability and aging. In yeast, sirtuins promote the extension of life span while in mammals they mediate the beneficial and anti-aging effects of physical exercise and caloric restriction [12].

Sirtuins have been recognized as pivotal regulators of cellular stress responses [13]. In response to a broad range of stress stimuli, they contribute to the adaptation of cellular physiology to harsh and varying conditions ensuring the maintenance of cellular homeostasis [14]. Stress stimuli promptly regulate expression of sirtuins and modulate their activation mainly by controlling their post-translational modification [15–17]. Sirtuins act mainly via two different mechanisms: (i) they promote heterochromatin formation at different chromosomal loci. This is achieved either by direct deacetylation of histones or by modulation of the activity and/or the recruitment of other histone modifiers such as acetyltransferases and methyltransferases [18]; (ii) sirtuins control numerous chromatin unrelated targets mainly via direct deacetylation. These targets mostly comprise enzymes and transcription factors such as p53, FOXO, MyoD, and NF-κB. In some cases, the same molecular targets are addressed by different sirtuins [18] (Fig. 1b). Finally, recent studies demonstrated the presence of a mutual regulation between different members of mammalian sirtuins, indicating that these molecules are part of a highly complex molecular network that maintains cellular homeostasis [19–22].

## Sirt1 in the Development of Cardiovascular System: Role in Congenital Heart Defects

Congenital heart diseases (CHDs) represent the most common category of birth defects with a prevalence of around 1% in the global population. Recent advances in the early diagnosis, corrective surgery, and post-operatory care have significantly reduced mortality in affected children, substantially increasing the percentage of patients surviving into adulthood [23]. Although several genetic aberrations have been associated with CHDs, the majority of cases does not depend on monogenetic causes [24]. Certain maternal conditions such as diabetes, obesity, hypertension, and exposure to toxic substances have been recognized as risk factors associated with CHDs [24]. The molecular pathways that underlie the development of pathological cardiovascular conditions during pregnancy and after birth as well as the influence of environmental factors are still not well understood.

**Fig. 2:**
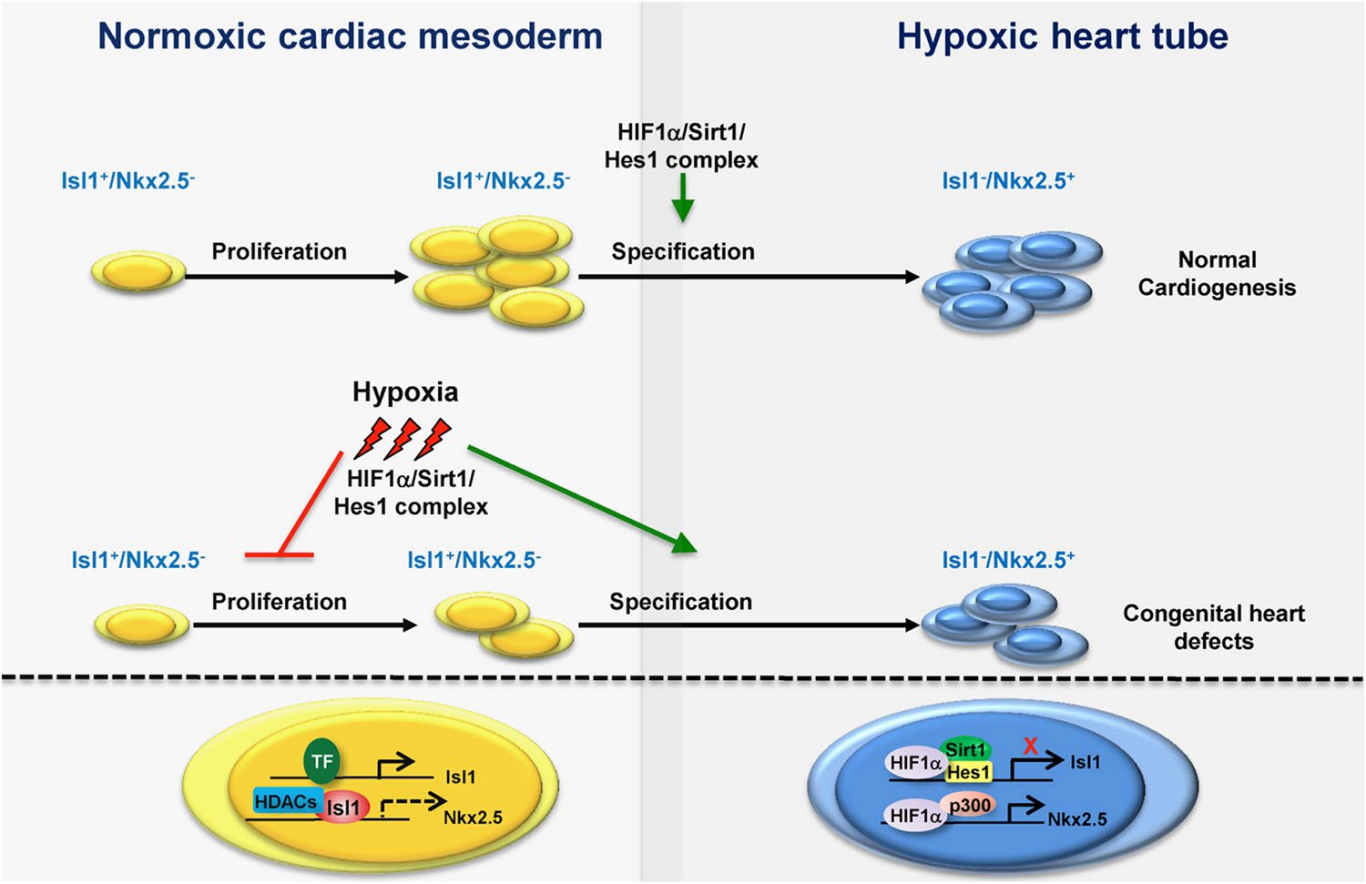
Sirt1 regulates proliferation and differentiation of CPCs in the SHF under physiological conditions and instigates aberrant cardiogenesis in response to hypoxia. In CPCs of the SHF (yellow), ISL1 recruits histone deacetylases (HDACs) to the promoter of the *Nkx2.5* gene, inhibiting its expression and thereby promoting ISL1^+^ cells expansion. As ISL1^+^ cells incorporate into the hypoxic heart tube, Sirt1 stimulates the commitment of these cells toward the cardiomyocyte lineage (blue) by forming a molecular complex with HIF1α and the transcription factor HES1 at the *Isl1* gene promoter thereby epigenetically inhibiting *Isl1* expression. This process promotes *Nkx2.5* expression and cardiomyocyte differentiation. Pathological exposure to hypoxia promotes formation of a HIF1α/Sirt1/Hes1 complex at the *Isl1* promoter leading to the premature specification of CPCs, causing congenital heart defects [26]

Growing experimental evidence supports an important role of Sirt1 in the development of cardiovascular system. Sirt1 is highly expressed in the embryonic heart but its expression dramatically declines in adult animals [10]. Sirt1 constitutive knockout animals show congenital cardiac abnormalities and die mostly before the age of 2 weeks after birth (Table 1) [25, 26]. Recently, we uncovered a novel role of Sirt1 in the regulation of cardiac progenitor cell (CPC) proliferation and specification in dependence on oxygen availability (Fig. 2). Although hypoxia was described as a risk factor for CHD, the molecular mechanisms remained largely unknown [26]. We discovered that during early stages of heart morphogenesis spatial and temporal differences in oxygen concentration determine expression of the critical transcription factors, ISL1 and NKX2.5, thereby regulating CPCs proliferation and specification. Moreover, we have demonstrated that Sirt1 is a critical factor, which translates differences in oxygen concentration into transcriptional responses [26].

**Table 1:** Role of sirtuins in the cardiovascular system

Sirtuin	Cardiac phenotype in mouse models under basal conditions	Role in response to stress stimuli	Gene alterations associated with cardiovascular diseases in humans
Sirt1	• Congenital heart abnormalities in constitutive KO mice [25, 26] • Conductive disturbances, insulin resistance and cardiac hypertrophy in cardiac-specific KO mice [31, 44] • Low to moderate expression in Tg-mice inhibits age-dependent cardiac remodeling [53]	• Cardiomyocytes survival [45–50]	• SNPs at the Sirt1 promoter associate with ventricular septal defects [33] • SNPs correlate with acute myocardial infarction [88]
		*Adverse effects* • Cardiac abnormalities in response to maternal exposure to hypoxia [26] • Promotes mitochondrial dysfunctions in failing hearts [52] • Promotes cardiac hypertrophy in response to pressure overload [51]	
	*Adverse effects* • High levels of expression in Tg-mice promote age-dependent fibrosis and hypertrophy [53]		
Sirt2	• Increased age-dependent cardiac remodeling in constitutive KO mice [54]	• Inhibition of hypertrophy [54]	• SNPs correlate with acute myocardial infarction [87]
Sirt3	• Increased cardiac remodeling in constitutive KO mice [60, 63, 64]	• Inhibition of cardiac remodeling and maintenance of mitochondrial functions [60–68]	• SNPs correlate with acute myocardial infarction [89] • SNPs correlate with PAH [101]
Sirt4	• No obvious phenotype in constitutive KO mice [69]	*Adverse effects* • Promotes pathological cardiac hypertrophy in response to pressure overload [69]	Not reported
Sirt5	• Increased cardiac remodeling in constitutive KO mice [70]	• Maintains mitochondrial functionality and prevents adverse remodeling [71, 72]	Not reported
Sirt6	• Increased cardiac remodeling in constitutive KO mice [73]	• Inhibits pro-hypertrophic and pro-fibrotic pathways [73–82]	• SNPs correlate with acute myocardial infarction [90]
Sirt7	• Age-dependent cardiac hypertrophy, fibrosis and inflammatory cardiomyopathy [83, 84]	*Adverse effects* • Prevents fibrosis and scar formation in response to ischemia reperfusion injury [85]	Not reported

The table summarizes the major roles of sirtuins in the heart under basal conditions or in response to exposure to stress stimuli. The table also reports known alterations of sirtuin genes, which correlate with cardiac diseases in human patients (*Tg-mice* transgenic mice, *SNPs* single-nucleotide polymorphisms, *KO* knock-out, *PAH* pulmonary arterial hypertension) [25, 26, 31, 33, 44–54, 60–85, 87–90, 101]

The heart is built from cells either located in the first heart field (FHF) or the second heart field (SHF) [27, 28]. The FHF forms the primary heart tube and parts of the left ventricle, whereas CPCs from the SHF contribute to the atria, the right ventricle, and the venous and anterior pole of the heart [29, 30]. After expression in cells that give rise to both the FHF and SHF, ISL1 is primarily found in CPCs of the SHF, where it is involved in the regulation of cellular proliferation. NKX2.5 does not show a heart field-specific expression but seems to be expressed at later stages of cardiomyogenic differentiation when CPCs start to turn off the expression of ISL1 and are acquiring a more mature cardiomyocyte phenotype [29, 30]. In the SHF, ISL1 inhibits premature differentiation of CPCs by recruiting histone deacetylases (HDACs) to the *Nkx2.5* promoter, leading thereby to inhibition of NKX2.5 expression. This process permits expansion of ISL1^+^ cells [26]. Interestingly, the particular niche where ISL1^+^ cells reside maintains relatively high oxygen concentration. In contrast, regions of the developing heart tube, where NKX2.5 activity leads to CPCs specification and subsequent differentiation into cardiomyocytes, are hypoxic (Fig. 2). In the forming heart, the reduced oxygen concentration triggers recruitment of the hypoxia inducible factor HIF1α to the *Isl1* promoter. HIF1α then attracts the transcription factor HES1 and Sirt1, resulting in Sirt1-mediated epigenetic silencing of ISL1 expression. At the same time, the inhibitory effect of ISL1 on the *Nkx2.5* promoter is relieved, NKX2.5 expression increases, thereby ensuring commitment of CPCs to the cardiomyocyte lineage [26, 29]. We have further demonstrated that the exposition of female mice to pathological hypoxia leads to precocious specification of ISL1^+^ cells in developing embryos due to aberrant recruitment of SIRT1 to the *Isl1* promoter and inactivation of *Isl1* expression. As a consequence of expression changes induced by exposure to the pathological hypoxia, the pools of proliferating and committed CPCs are abated and congenital heart defects develop (Fig. 2). Inactivation of Sirt1 specifically in ISL1^+^ cells of the SHF is able to avert the negative effects of pathological hypoxia during heart development, preventing the onset of cardiac abnormalities [26]. Interestingly, inactivation of Sirt1 in ISL1^+^ cells under physiological conditions does not result in major cardiac abnormalities, although Isl1 expression is promoted and Nkx2.5 expression is decreased, indicating that under physiological conditions still unknown mechanisms compensate for Sirt1 deficiency in the SHF [26].

Another important function of Sirt1 in cardiac physiology is the regulation of cardiac electrical activity. Sirt1-dependent deacetylation of the voltage-gated Na^+^ channel, Na_v_1.5, at lysine 1479 is a prerequisite for its correct localization in the cell membrane. Cardiomyocyte-specific deletion of Sirt1 in mice causes cardiac conductive abnormalities and premature death due to arrhythmia (Table 1) [31]. Interestingly, mutations in the SCN5A gene coding for NA_v_1.5 have been found in patients suffering from arrhythmia disorders such as the long-QT and the Brugada syndrome and other inherited conduction diseases [32]. Such arrhythmias often affect pediatric patients and underlie many causes of sudden death in pediatric population.

Taken together, there is strong experimental evidence that Sirt1 is important for proper heart development and maintenance of cardiac functions. Therefore, changes in Sirt1 expression and/or activity may be associated with the development of CHDs. At this point, it is worth noting that an occurrence of single-nucleotide polymorphism (SNPs) at the Sirt1 promoter, which might alter Sirt1 expression, was found in human subjects with ventricular septal defects (Table 1) [33]. Furthermore, reduction of the activity and/or expression of Sirt1 and Sirt3 in different fetal tissues has been described in maternal conditions favoring occurrence of congenital heart diseases, such as gestational diabetes and obesity [34–37]. Moreover, mutations in genes encoding enzymes from the NAD biosynthesis pathway have been associated with congenital heart malformations [38]. Since NAD is a fundamental co-enzyme required for sirtuin activity, it is reasonable to assume that any disturbances in NAD availability might inhibit sirtuin activity and result in a cardiac phenotype.

## Role of Sirtuins in Cardiac Stress Responses

The heart is a very dynamic organ that can adapt its function in response to different physiological and pathological stimuli. During adaption, the heart activates a complex network of metabolic and molecular changes that enable cardiac remodeling [39]. Cardiac remodeling occurs under physiological conditions such as physical exercise or changes in metabolism. In response to persistent stress, however, cardiac remodeling may promote deterioration of ventricular function leading to heart failure. Adverse cardiac remodeling processes comprise cardiomyocytes hypertrophy or death, myocardial fibrosis and inflammation [40, 41]. Mitochondria not only play a pivotal role in the maintenance of cardiac functions by providing energy but also contribute to oxidative stress. Mitochondrial dysfunction is an important event that contributes to cardiac remodeling and heart failure as a consequence of prolonged exposure of the heart to challenging stimuli [42]. Several other molecular pathways are associated with adverse cardiac remodeling. For instance, the differentiation of cardiac fibroblasts into myofibroblasts is a crucial event that leads to cardiac fibrosis further compromising heart contractility and contributing to heart failure [43].

Sirtuins have been shown to contribute to homeostasis of the heart under physiological and stress conditions by controlling a vast number of molecular pathways. Sirt1 promotes the maintenance of mitochondrial homeostasis by activating key transcription factors involved in biogenesis of mitochondria. Consequently, mitochondrial biogenesis and function are impaired in hearts of cardiac-specific Sirt1 knockout mice. Altogether, inactivation of Sirt1 in cardiomyocytes led to symptoms characteristic for diabetic cardiomyopathy (DCM) including cardiac hypertrophy, abnormal glucose metabolism and insulin resistance. Importantly, treatment of DCM mice with a Sirt1 activator, resveratrol, reverses the DCM phenotype [44]. Several further investigations reported protective effects of Sirt1 in the heart, especially under stress conditions. Generally, Sirt1 lowers oxidative stress and favors cardiomyocytes survival [45–50]. Surprisingly, however, it was observed that Sirt1 KO mice were protected from cardiac hypertrophy in response to pressure overload [51]. Furthermore, Sirt1 is upregulated in failing hearts where it promotes mitochondrial dysfunction and heart failure [52]. These controversial findings suggest that Sirt1 might exert protective or deleterious effects in the heart probably depending on the type of stress that dominates. In addition, the influence of Sirt1 on cardiac physiology appears to be dosage-dependent. In fact, high levels of Sirt1 (more than 12 times above the normal level) have detrimental effects on the cardiac function, while a low to moderate overexpression of Sirt1 in transgenic mice inhibits age-dependent development of cardiac hypertrophy and fibrosis [53]. These data illustrate the high degree of complexity of Sirt1-dependent regulatory processes during cardiac stress responses (Table 1).

Much less is known about the role of the cytoplasmic Sirt2 in the heart. One study described a protective role of Sirt2 by preventing age-associated and stress-induced cardiac remodeling in mice through activation of anti-hypertrophic signaling pathways (Table 1) [54]. Sirt2 has been also shown to maintain mitochondrial homeostasis, protect against oxidative stress and improve insulin sensitivity in hepatocytes [55]. However, it is not known whether similar mechanisms might contribute to cardiac homeostasis.

Mitochondrial sirtuins, Sirt3, Sirt4, and Sirt5, attracted more attention than Sirt2 in the heart. Sirt3 regulates the global acetylome of mitochondrial proteins. Inactivation of Sirt3 results in a significant increase in the acetylation levels of key mitochondrial enzymes and in a specific inactivation of the mitochondrial complex I activity, suggesting that Sirt3 is an important factor maintaining basal ATP levels [56–60]. Sirt3-deficient mice show enhanced cardiac fibrosis and hypertrophy, which manifest already in young animals and progress with aging. In addition, Sirt3 knockout mice display higher rate of cardiac remodeling and cardiac dysfunction in response to stress stimuli due to the impaired mitochondrial function and accumulation of oxidative stress. In contrast, Sirt3 overexpressing mice are protected against cardiac stressors [60–64]. Several other reports demonstrated an essential role for Sirt3 in cardiac protection under different stress conditions. Sirt3 overexpression in primary cardiomyocytes reduced cellular levels of reactive oxygen species (ROS) by deacetylating the transcription factor Foxo3a and thereby stimulating the expression of antioxidant enzymes-encoding genes. Through decreased ROS level and other mechanisms, Sirt3 further contributed to lower apoptosis in response to stress [63, 65–67]. Moreover, Sirt3 prevents cardiac differentiation of fibroblasts to myofibroblasts and hence fibrosis in vitro [68]. In contrast to the beneficial effects of Sirt3, Sirt4 has been shown to promote pathological cardiac hypertrophy in response to pressure overload by enhancing oxidative stress. Interestingly, Sirt4 seems to antagonize Sirt3-dependent activation of an antioxidative enzyme, the manganese dependent superoxide dismutase (MnSOD) [69]. The last mitochondrial sirtuin, Sirt5, exhibits predominantly a protective role in the heart. Sirt5 was shown to desuccinylate and activate enzymes involved in fatty acid oxidation. Remarkably, the main enzyme regulated by Sirt5 in the heart is ECHA. Decreased ECHA activity was identified in the heart of Sirt5 knockout mice as a direct cause responsible for progressive cardiac dysfunction starting already in young animals and culminating in loss of ATP reservoirs and enhanced cardiac hypertrophy [70]. Two further reports demonstrated that Sirt5 protects the heart from injuries such as ischemia reperfusion and pressure overload by preserving mitochondrial function (Table 1) [71, 72].

Cardiac function is also strongly influenced by the nuclear sirtuin, Sirt6. Cardiomyocyte-specific Sirt6 KO animals develop spontaneous cardiac hypertrophy already at around 8–12 weeks of age [73]. Moreover, Sirt6 downregulation has been observed in failing human hearts and in mice exposed to hypertrophic stimuli while transgenic mice overexpressing Sirt6 were protected from hypertrophy in response to stress [73]. Sirt6 has been shown to inhibit different pro-hypertrophic pathways such as IGF-Akt, NF-κB and STAT3 and prevent oxidative stress and cardiac fibrosis through inhibition of the NF-κB and the AMPK/angiotensin-converting enzyme 2 pathways [73–81]. Furthermore, Sirt6 exerts a protective role in the heart by maintaining telomeres integrity in response to pressure overload (Table 1) [82].

Finally, Sirt7 plays a crucial role in the maintenance of heart homeostasis. Sirt7 KO mice show higher age-dependent accumulation of cardiac hypertrophy, fibrosis, inflammatory cardiomyopathy and cardiomyocyte apoptosis as compared with wild type littermates [83, 84]. This phenotype might derive from increased activation of hypertrophic pathways and p53 [84]. Another study revealed that Sirt7 deacetylates and activates the transcription factor GABPβ-1, a master regulator of the transcription of nuclear-encoded mitochondrial genes, and thus promotes proper mitochondria biogenesis [83]. The authors argue that an impaired mitochondrial function contributes to cardiac dysfunction and hypertrophy observed in Sirt7 knockout mice. Notably, Sirt7 also negatively affects cardiac function: In response to cardiac injury, Sirt7 KO animals show reduced fibrosis and impaired scar formation that often results in cardiac rapture [85]. Sirt7 stimulates fibrosis by stabilizing the TGFß-receptor-1 through inhibition of autophagy [85]. Interestingly, stimulation of fibrosis takes place only in young animals after myocardial infarct induction. In old Sirt7 knockout animals, an increase in age-dependent fibrosis was observed [84]. Such functional duality may be explained by the fact that cardiac fibrosis in response to injury and during aging depends on the activation of different molecular pathways (Table 1) [85].

At this point, it should be also mentioned that sirtuins have also been extensively implicated in maintenance of endothelial cell homeostasis through inhibition of inflammation and regulation of numerous other molecular pathways. Through these mechanisms, sirtuins prevent endothelial dysfunction and development of atherosclerosis [86]. Although a comprehensive discussion of the role of sirtuins in endothelial cells is beyond the scope of this review, we would like to point out that most studies indicate a protective role of sirtuins in the vascular system. The importance of sirtuins in the cardiovascular system was additionally strengthened by the description of polymorphisms in the Sirt1, Sirt2, Sirt3, and Sirt6 promoters in patients with acute myocardial infarction [87–90].

## Sirtuins: Possible Targets in Pediatric Cardiology?

Although the etiology of heart diseases might differ between children and adult patients, in certain cases, as for example adverse cardiac remodeling, the same pathological mechanisms are employed [91–93]. As described above, growing experimental evidence supports a role of Sirt1 in the development of congenital heart diseases. Still, very little is known about the role of other sirtuins in cardiac diseases during infancy. Inactivation of other members of the sirtuin family in mice leads to cardiac dysfunctions that mainly manifest with aging or in response to stress stimuli. Nevertheless, impaired activity of sirtuins might also be associated with deterioration of cardiac functions in children. Sirtuins are likely involved in metabolic diseases caused by genetic alterations of mitochondrial genes, which lead to dysfunctions of the respiratory chain. These diseases are often associated with skeletal myopathy and cardiomyopathies such as conduction defects and hypertrophic cardiomyopathy [94]. Interestingly, it has been estimated that cardiomyopathy occurs in 20–40% of these patients already during childhood [95]. Since sirtuins residing in mitochondria (Sirt3, Sirt4, and Sirt5), but also nuclear sirtuins, strongly affect mitochondrial functions, they are probably also involved in the cellular responses to mitochondrial dysfunctions. Conversely, mitochondrial defects will affect the activity of sirtuins, since mitochondria constitute a major source for oxidation of NADH and mitochondrial malfunction can result in an unbalanced ratio of NAD^+^ and NADH.

Friedreich’s ataxia (FRDA) is an autosomal recessive early onset (mean age of onset between 10 and 15 years) disorder associated with dysfunctional assembly of the mitochondrial respiratory chain. Patients manifest with different multisystem alterations and in 85% of the cases die as a consequence of heart failure [96]. In a mouse model of FRDA, it has been demonstrated that mitochondrial dysfunction is associated with reduced levels of NAD, which led to impaired Sirt3 activity [97]. Normalization of the unbalanced ratio of NAD^+^/NADH in FRDA mice restores cardiac function in a Sirt3-dependent fashion, suggesting that Sirt3 might constitute a possible target to ameliorate cardiac functions in FRDA patients [98]. Interestingly, hyperacetylation of mitochondrial enzymes has also been observed in other respiratory chains defects, suggesting that inactivation of Sirt3 might play a role in cardiomyopathies caused by mitochondrial dysfunctions [97]. In support of the significance of sirtuin function in mitochondria-based diseases, downregulation of the NAD^+^ as well as Sirt1, Sirt3, and Sirt4 levels has been described in human skin fibroblasts of patients with cytochrome c-oxidase deficiency, which also manifests with cardiac dysfunctions [99]. Currently, it is not known whether hearts of patients with cytochrome c-oxidase deficiency show reduced sirtuin activities and whether stimulation of sirtuin activities might ameliorate cardiomyopathy.

**Fig. 3:**
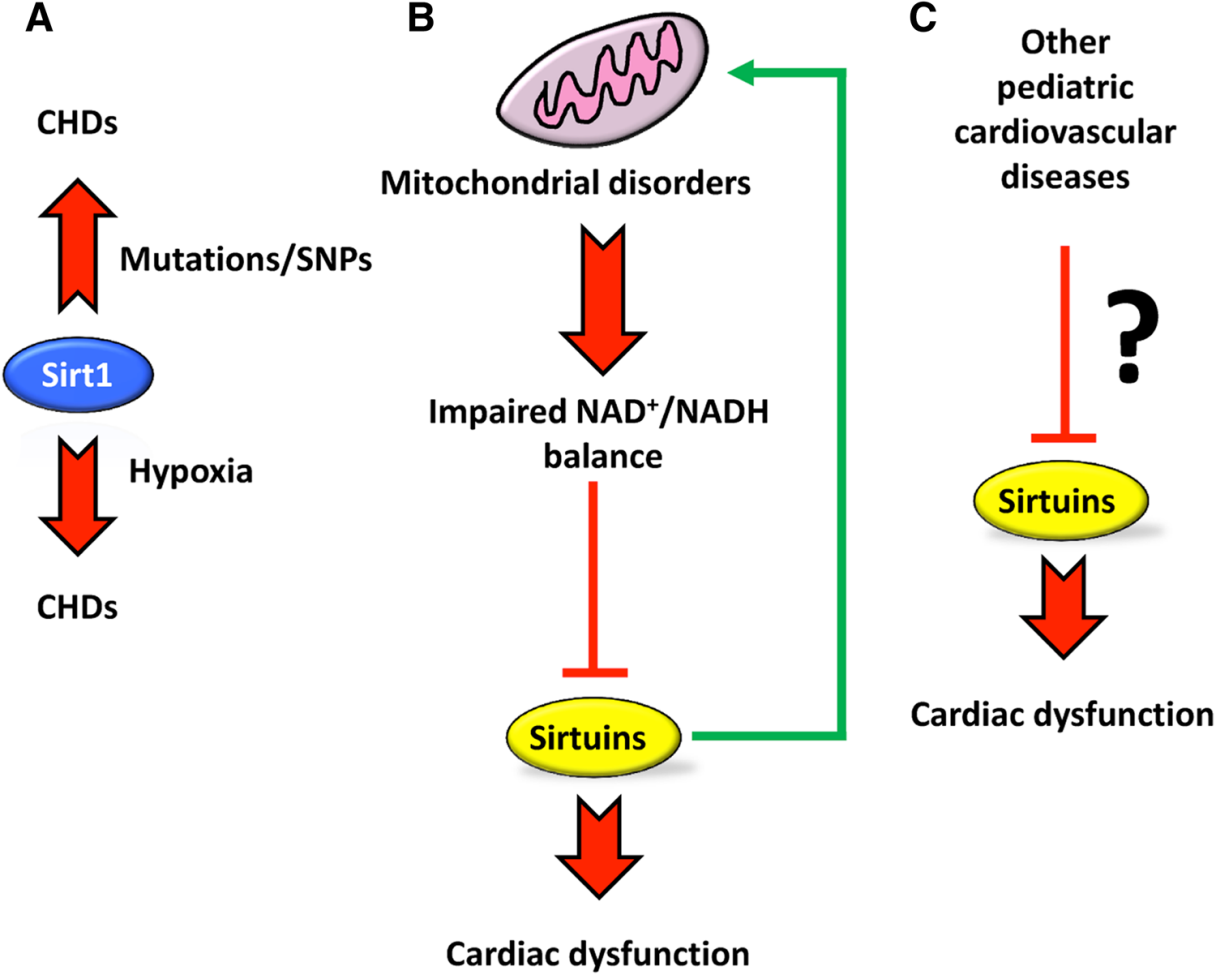
Possible roles of mammalian sirtuins in pediatric cardiology. **a** Sirt1 plays a pivotal role in normal cardiac development. Lost or reduced Sirt1 function due to mutations or SNPs may lead to congenital heart diseases (CHDs). Moreover, Sirt1 is responsible for the onset of CHDs in response to pathological exposure to hypoxia [25, 26, 31]. **b** Mitochondrial disorders may result in impaired NAD^+^/NADH ratio that can lead to inhibition of sirtuins resulting in more severe cardiac dysfunctions [97, 98]. Sirtuins can also directly improve mitochondrial functions. **c** Altered functions of sirtuins may contribute to accelerated deterioration of heart physiology also in other cardiac diseases, which manifest in the childhood

Pharmacological manipulation of sirtuins might be used to delay the onset of cardiac dysfunction in several diseases. For instance, Sirt1 activation has been shown to ameliorate cardiac hypertrophy and fibrosis and restore cardiac diastolic function in dystrophic mice [100]. Furthermore, Sirt3 deficiency in mice has been associated with development of pulmonary arterial hypertension (PAH), which causes right ventricular hypertrophy. Interestingly, a strong association of SNPs polymorphism in the Sirt3 gene, that might affect its expression or function, has been found in patients suffering from several forms of idiopathic PAH [101]. Hence, stimulation of Sirt3 activity might be seen as a potential new means to treat PAH. A scheme depicting the potential roles of different sirtuins in the pathogenesis of cardiac diseases is shown in Fig. 3 (Table 1).

## Conclusions

The molecular pathways that govern cardiac diseases need to be investigated more thoroughly in children and young adults. Sirtuins are important regulators of cardiac functions but their role in pediatric cardiac diseases just starts to emerge. The experimental evidence summarized in this review suggests that pharmacological manipulation of sirtuins represents an attractive option to develop new therapies that might improve heart function in children suffering from different heart diseases.
